# Use of GPS Tracking Technology to Measure Mobility in Older Adults: A Systematic Review

**DOI:** 10.1093/geroni/igaa057.636

**Published:** 2020-12-16

**Authors:** Jane Chung, Lana Sargent, Roy Brown

**Affiliations:** 1 Virginia Commonwealth University School of Nursing, Richmond, Virginia, United States; 2 Virginia Commonwealth University, Richmond, Virginia, United States

## Abstract

Global positioning system (GPS) tracking technology is increasingly used in aging research to objectively measure the spatial and temporal aspects of mobility in older adults. The review aims to systematically synthesize the literature to identify GPS-driven mobility measures and potential determinants of mobility limitation for community-dwelling older adults. A systematic search of six electronic databases was conducted. A total of 4897 articles were found with 2578 left to review after deduplication. Twenty-five studies met inclusion criteria: 24 cross-sectional studies and one follow-up study that measured mobility changes over time. Various types of GPS devices were used, including smartphones, GPS watches, or portable data logging kits. The GPS tracking period ranged from 1 to 30 days. The daily device wear time varied from 10 to 24 hours. Commonly reported GPS-based mobility measures included time out of home, distance moved, the number of out-of-home trips or walking tracts, the number of visited places, life-space area, and walking speed. Twenty-one studies reported some aspects of demographic, physical, psychosocial, or environmental factors related to the levels of GPS-based mobility. GPS tracking technology can continuously record individuals’ activities and functional abilities within their life space. We found that there was heterogeneity in ways of applying GPS technology and defining and measuring mobility in community-dwelling older adults. Given the lack of consistency in GPS-based mobility assessment, a clear definition of mobility and standardization of GPS data collection and analysis are required for comparison across studies and better understanding determinants of mobility limitation in community-dwelling older adults.

